# Supplementary material for: The cerebellum regulates fear extinction through thalamo-prefrontal cortex interactions in male mice
Source: Nat Commun. 2023 Mar 17;14:1508. doi: 10.1038/s41467-023-36943-w (PMC10023697; doi:10.1038/s41467-023-36943-w)
Supplement: Supplementary file 1 — Supplementary Information [file 41467_2023_36943_MOESM1_ESM.pdf]

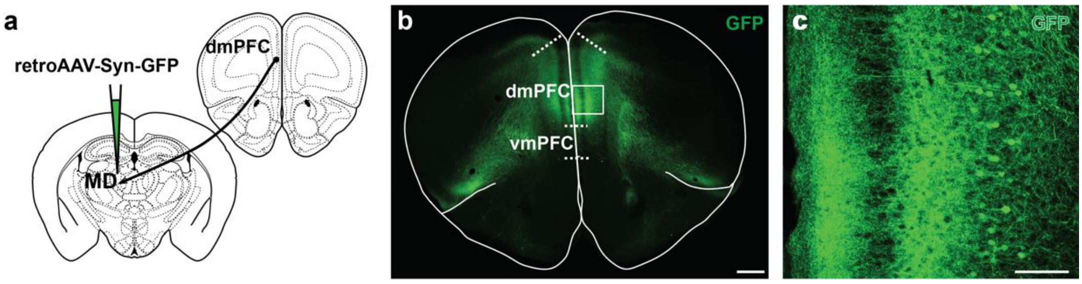

Supplementary Fig. 1. dmPFC neurons project to the MD. a, Retrograde tracing strategy by injection of retrograde AAV-GFP in the MD. **b,** dmPFC-MD projecting neurons expressing retrograde AAV-GFP (green, scale bar, 500 µm). **c,** Zoom-in of dmPFC section from (**b**), GFP+ neurons in cortical layers of the dmPFC projecting to MD (scale bar, 100 µm). n=3 replicates. Brain schematic in panel (a) modified from the Allen Mouse Brain Atlas and Allen Reference Atlas – Mouse Brain[73,74] http://atlas.brain-map.org/atlas?atlas=1#atlas=1&plate=100960384, http://atlas.brain-map.org/atlas?atlas=1#atlas=1&plate=100960136, http://atlas.brain-map.org/atlas?atlas=1#atlas=1&plate=100960240.

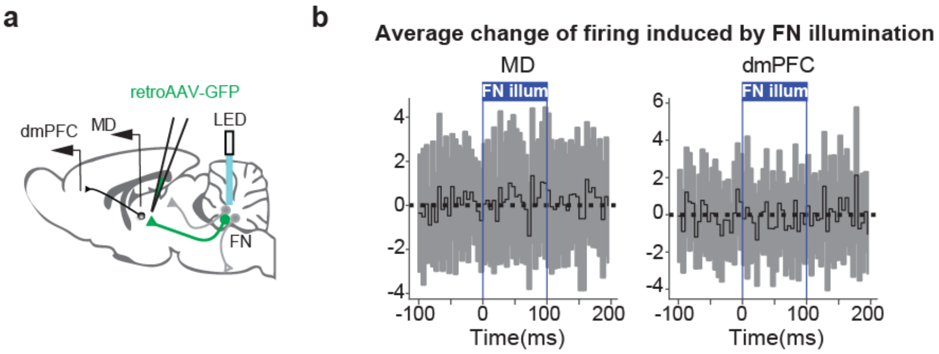

Supplementary Fig. 2. Cell responses to FN illumination in absence of ChR2 expression. a, Strategy used for the FN illumination control experiment, representing the local injection of retrograde AAV-eGFP in the MD and implantation of recording electrodes in MD and dmPFC. **b**, PSTH (5 ms bins) displaying the change in firing rate (average ± SD) during 100 ms illumination of the FN in MD (left), and in dmPFC (right). The light stimulation is represented by a blue rectangle.

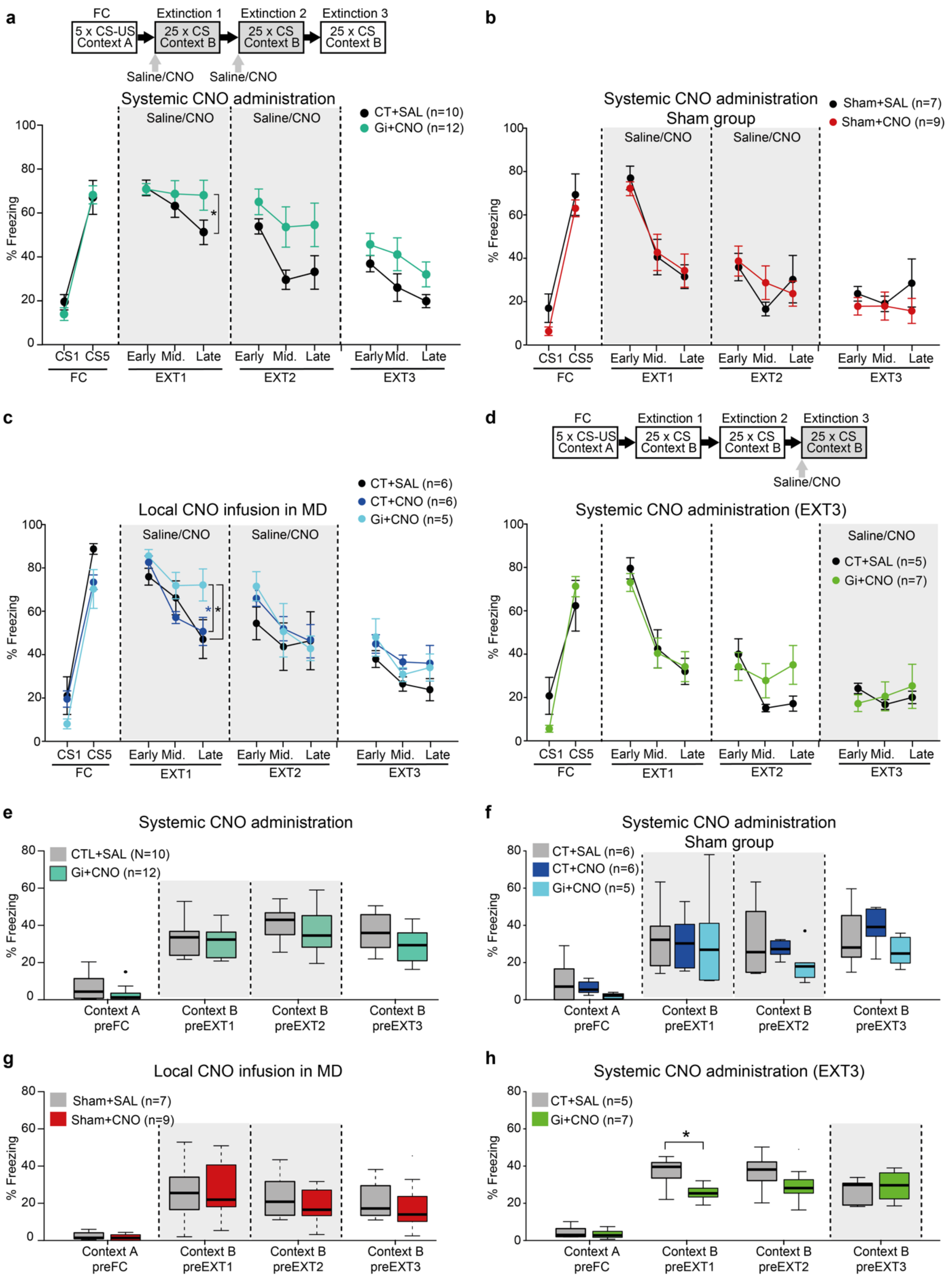

Supplementary Fig. 3. Specific chemogenetic inhibition of FN terminals in MD modulates fear extinction learning. a, Top panel: classical fear conditioning and extinction protocol used, with Saline or CNO administration during extinctions 1 and 2. Bottom: same data as Fig. 4e without separating high-freezing and low-freezing mice; a reduction of fear extinction is still visible in EXT1. Lines represent mean ± SEM. Posthoc t-test, * $p<0.05$. **b**, Verification that the CNO dose used has not effect on freezing in absence of DREADD-Gi expression (Sham mice); no differences in freezing levels were found between sham mice injected i.p. with CNO or saline (Sham+SAL, n = 7; Sham+CNO, n = 9). Posthoc t-test, $p > 0.5$. **c,** Specific inhibition of FN-terminals in MD by intracranial CNO infusion in EXT1 and EXT2 sessions (Gi+CNO, n = 5) reduced extinction of fear

response compared to the control groups (CT+SAL, n = 6; CT+CNO, n = 6). **d,** Top panel: fear conditioning and extinction protocol with Saline or CNO administration in EXT3 in mice expressing inhibitory DREADD in FN neurons projecting to MD. Inhibition of FN input to MD during EXT3 (Gi+CNO, n = 7) did not affect the expression of CS-freezing response compared to control group (CT+SAL, n = 5). Lines represent mean ± SEM. Posthoc t-test, p>0.5 Holm-Sidak corrected. **e-h** baseline freezing levels before FC (context A) and before each extinction session (context B). A small difference between groups was observed in the EXT1 baseline freezing for the late inhibition experiment (**h**); this reveals a small heterogeneity between the groups of animals which, at that time of the protocol received both the same treatment; however the inter-group difference in baseline freezing fell below significance during EXT2 (and EXT3). Posthoc t-test, * p<0.05. Boxplots represent quartiles and whiskers correspond to range; points are singled as outliers if they deviate more than 1.5 x interquartile range from the nearest quartile. Data available at doi:10.5061/dryad.9kd51c5ng All tests are two-sided. Detailed statistical results are available in the Supplementary Tables.

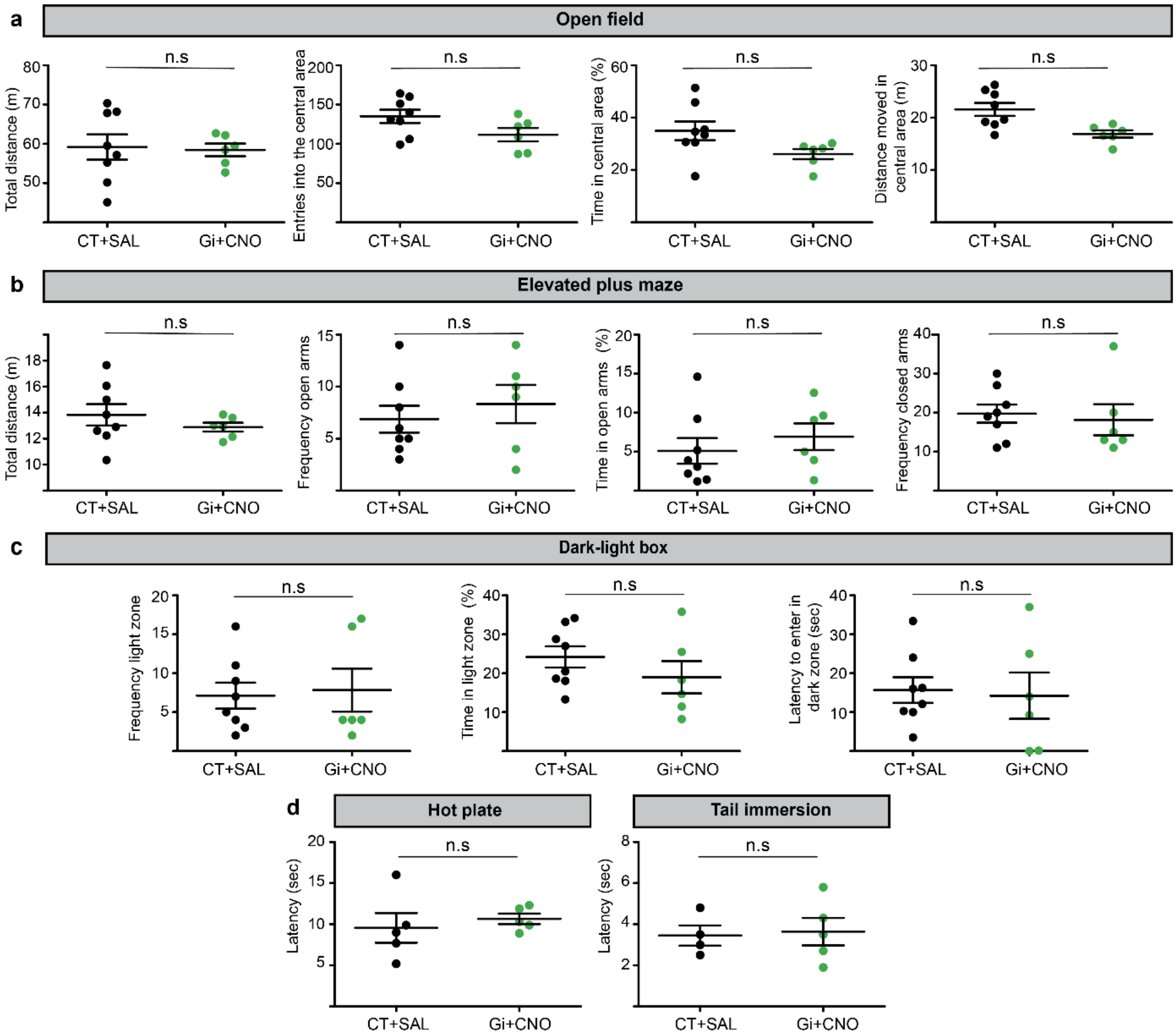

Supplementary Fig. 4. FN input to MD does not contribute either to anxiety like behavior or nociception. a-c, FN-MD chemogenetic inhibition (Gi+CNO, n = 6) had no effect on anxiety like behavior compared to the control mice (CT+SAL, n = 8) in the open field (**a**), in the elevated plus maze (**b**), or in the dark-light box (**c**). **d,** Inhibition of FN-MD projections had not significant effect on hot plate (Gi+CNO, n = 5; CT+SAL, n = 5) (left) or tail immersion (Gi+CNO, n = 4; CT+SAL, n = 4) (right) tests compared to the control. Scatter dot plot with mean ± SEM, n.s.: Mann-Whitney U test, p > 0.05. Data available at doi:10.5061/dryad.9kd51c5ng . All tests are two-sided. Detailed statistical results are available in the Supplementary Tables.

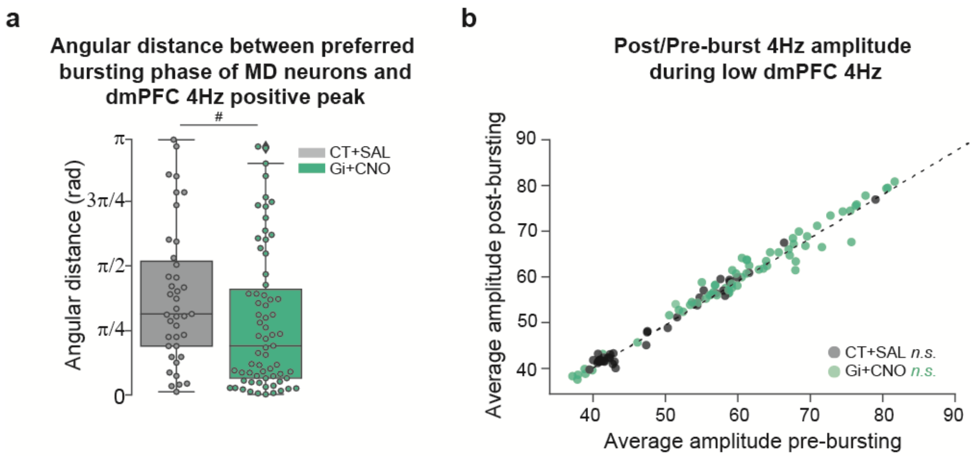

Supplementary Fig. 5. The inhibition of FN inputs to MD induces a greater phase locking to dmPFC 4Hz positive peaks and MD bursting is not associated to an increase in 4Hz amplitude during periods of low dmPFC 4Hz. a, Distributions of angular distance between referential bursting phases of MD neurons and pi (positive peaks in dmPFC 4Hz oscillations). Mann-Whitney U test, #p=0.01. **b**, The average 4 Hz amplitude of the 500 ms following MD bursting is not increased during episodes of low 4 Hz compared to the 500 ms preceding the burst. Wilcoxon test. All tests are two-sided. Detailed statistical results are available in the Supplementary Tables. Data available at doi:10.5061/dryad.9kd51c5ng

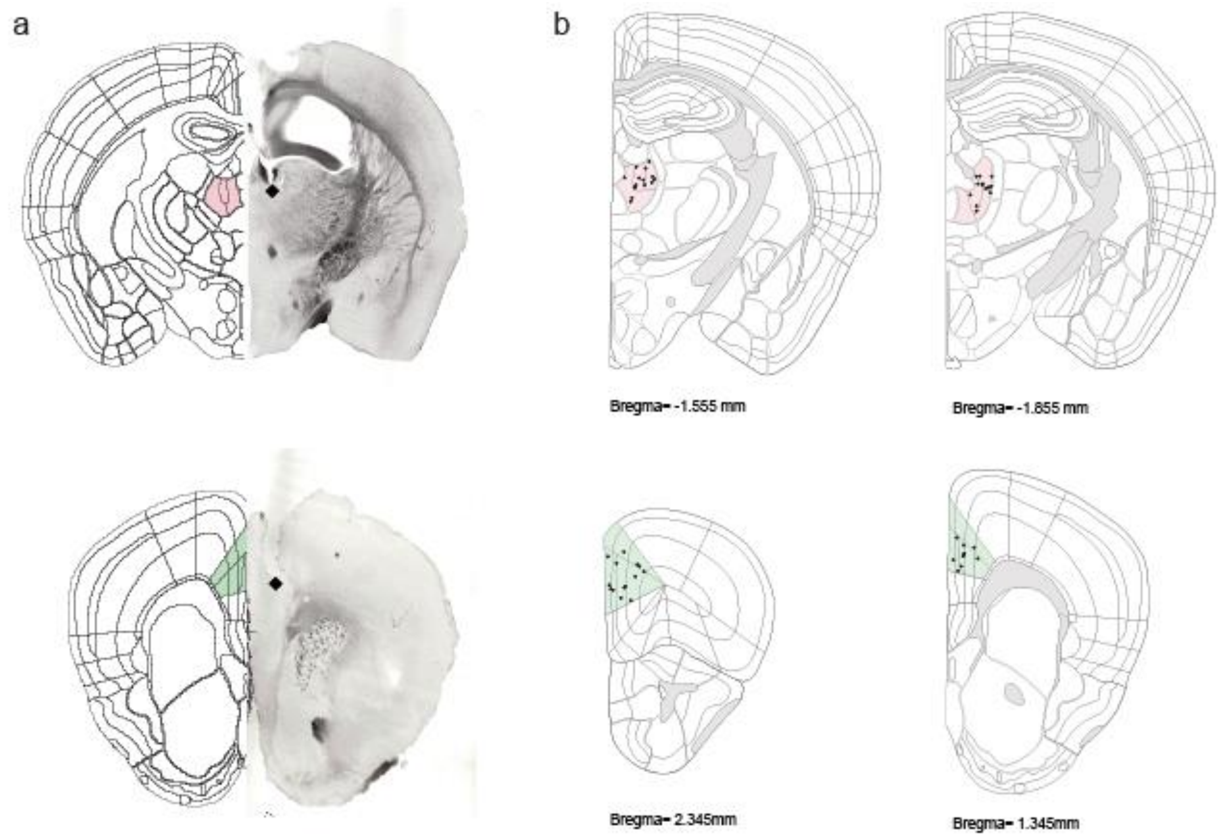

Supplementary Figure 6. Histological placement of the electrodes. a, example brain slices of MD (top) and dmPFC (bottom). An atlas schematic is provided on the left, where the the MD (pink) and dmPFC (green) are highlighted. The estimated tip of the bundle is labeled by the black losange. **b,** summary of the electrode position within the MD and dmPFC. All brain schematics are modified from the Allen Mouse Brain Atlas and Allen Reference Atlas – Mouse Brain[73,74] https://atlas.brain-map.org/atlas?atlas=1&plate=100960260 and http://atlas.brain-map.org/atlas?atlas=1#atlas=1&plate=100960365 for panel a, and https://atlas.brain-map.org/atlas?atlas=1&plate=100960244, https://atlas.brain-map.org/atlas?atlas=1&plate=100960232 https://atlas.brain-map.org/atlas?atlas=1&plate=100960360 https://atlas.brain-map.org/atlas?atlas=1&plate=100960360 for panel b.

Experiment	region	n cells	N mice
FN stimulation	MD	158.00	10.00
	dmPFC	36.00	7.00
FN-MD stimulation	MD	90.00	8.00
	dmPFC	48.00	8.00

Supplementary Table 1: Statistics for Optogenetic recordings.

Group	Group	Phase	region	n cells	N mice
Recorded neuron	CT+SAL	FC	MD	36.00	4.00
		EXT1	MD	39.00	4.00
		EXT3	MD	22.00	4.00
	Gi+CNO	FC	MD	49.00	5.00
		EXT1	MD	67.00	5.00
		EXT3	MD	51.00	5.00

Supplementary Table 2: Statistics for Chemogenetic recordings.

Group	Phase	region	n channels	N mice
CT+SAL	EXT1	MD	28.00	4.00
		dmPFC	14.00	4.00
Gi+CNO	EXT1	MD	30.00	5.00
		dmPFC	15.00	5.00

Supplementary Table 3: Statistics for LFP channels.

Value	Test	Structure	Statistic	p-value	Sig.
DeltaFR	One sample Wilcoxon test	MD	14.00	<0.001	***
DeltaFR	One sample Wilcoxon test	dmPFC	0.00	<0.001	***

Supplementary Table 4: Statistics for Fig 3f.

Mice	Phase	Effect	ANOVA	p-value	Sig.
High-freezing mice	FC	CS	F(1,8)=51.04	9.156e-05	***
		Treatment	F(1,8)=0.7107	0.4234	n.s.
		CS:Treatment	F(1,8)=0.01367	0.9098	n.s.
	EXT1	CS	F(2,18)=2.067	0.1556	n.s.
		Treatment	F(1,9)=5.952	0.03739	*
		CS:Treatment	F(2,18)=4.346	0.02884	*
	EXT2	CS	F(2,18)=4.637	0.02376	*
		Treatment	F(1,9)=8.956	0.01514	*
		CS:Treatment	F(2,18)=3.06	0.07178	n.s.
	EXT3	CS	F(2,18)=3.715	0.04459	*
		Treatment	F(1,9)=1.757	0.2177	n.s.
		CS:Treatment	F(2,18)=0.3146	0.734	n.s.
Low-freezing mice	FC	CS	F(1,8)=94.43	1.051e-05	***
		Treatment	F(1,8)=0.1369	0.721	n.s.
		CS:Treatment	F(1,8)=1.389	0.2725	n.s.
	EXT1	CS	F(218)=4.472	0.0265	*
		Treatment	F(19)=0.03038	0.8655	n.s.
		CS:Treatment	F(218)=0.5615	0.58	n.s.
	EXT2	CS	F(218)=12.88	0.0003373	***
		Treatment	F(19)=0.06678	0.8019	n.s.
		CS:Treatment	F(218)=0.2771	0.7611	n.s.
	EXT3	CS	F(218)=18.59	4.184e-05	*
		Treatment	F(19)=1.609	0.2365	n.s.
		CS:Treatment	F(218)=3.51	0.05161	n.s.

Supplementary Table 5: Statistics for Fig 4e ANOVAs.

Mice	Group 1	Group 2	Phase	CS period	Estimate (Grp1-Grp2)	SE	df	t	p-value	sig
High-freezing mice	Gi+CNO	CT+SAL	FC	CS1	7.41	10.91	16.00	0.68	0.51	n.s.
				CS5	5.60	10.91	16.00	0.51	0.61	n.s.
			EXT1	Early	1.83	8.30	23.84	0.22	0.83	n.s.
				Middle	-17.50	8.30	23.84	-2.11	0.05	*
				Late	-27.52	8.30	23.84	-3.31	0.00	**
			EXT2	Early	-18.81	13.76	15.75	-1.37	0.19	n.s.
				Middle	-49.02	13.76	15.75	-3.56	0.00	**
				Late	-37.85	13.76	15.75	-2.75	0.01	*
			EXT3	Early	-18.43	14.09	13.81	-1.31	0.21	n.s.
				Middle	-19.85	14.09	13.81	-1.41	0.18	n.s.
				Late	-11.63	14.09	13.81	-0.83	0.42	n.s.
Low-freezing mice	Gi+CNO	CT+SAL	FC	CS1	3.42	7.82	15.15	0.44	0.67	n.s.
				CS5	-7.97	7.82	15.15	-1.02	0.32	n.s.
			EXT1	Early	-0.67	9.26	16.69	-0.07	0.94	n.s.
				Middle	2.89	9.26	16.69	0.31	0.76	n.s.
				Late	-6.28	9.26	16.69	-0.68	0.51	n.s.
			EXT2	Early	-3.52	11.50	13.65	-0.31	0.76	n.s.
				Middle	1.01	11.50	13.65	0.09	0.93	n.s.
				Late	-5.46	11.50	13.65	-0.47	0.64	n.s.
			EXT3	Early	0.83	6.68	14.37	0.12	0.90	n.s.
				Middle	-10.47	6.68	14.37	-1.57	0.14	n.s.
				Late	-12.72	6.68	14.37	-1.91	0.08	n.s.
							9.00			

Supplementary Table 6: Statistics for Fig 4e contrasts.

Mice	Effect	ANOVA	p-value	Sig.
High-freezing mice	Treatment	F(1,9)=0.00802	0.93	n.s.
	Context	F(3,21)=63.65	0.00	***
	Treatment:Context	F(3,21)=1.607	0.22	n.s.
Low-freezing mice	Treatment	F(1,9)=2.532	0.15	n.s.
	Context	F(3,27)=36.52	0.00	***
	Treatment:Context	F(3,27)=3.189	0.04	*

Supplementary Table 7: Statistics for Fig 4f ANOVAs.

Mice	context	Group1	Group2	Estimate (Grp1-Grp2)	SE	df	t.ratio	p.value	Sig.
High-freezing mice	context A	CNO	SAL	-4.01	6.27	9.00	-0.64	0.54	n.s.
	context B preEXT1	CNO	SAL	-13.28	6.27	9.00	-2.12	0.06	n.s.
	context B preEXT2	CNO	SAL	0.87	6.79	9.00	0.13	0.90	n.s.
	context B preEXT3	CNO	SAL	-8.94	6.79	9.00	-1.32	0.22	n.s.
Low-freezing mice	context A	CNO	SAL	-6.02	4.56	9.00	-1.32	0.22	n.s.
	context B preEXT1	CNO	SAL	6.79	4.56	9.00	1.49	0.17	n.s.
	context B preEXT2	CNO	SAL	-9.77	4.56	9.00	-2.14	0.06	n.s.
	context B preEXT3	CNO	SAL	-8.24	4.56	9.00	-1.81	0.10	n.s.

Supplementary Table 8: Statistics for Fig 4f Contrasts.

Experiment	Phase	Effect	ANOVA	p-value	Sig.
EXT1 EXT2 systemic	FC	CS	F(1,18)=133.7	<0.001	***
		Treatment	F(1,18)=0.1936	0.665	n.s.
		CS:Treatment	F(1,18)=0.5587	0.464	n.s.
	EXT1	CS	F(2,40)=5.935	0.006	**
		Treatment	F(1,20)=1.538	0.2308	n.s.
		CS:Treatment	F(2,40)=3.38	0.04401	*
	EXT2	CS	F(2,40)=11.73	<0.001	***
		Treatment	F(1,20)=3.982	0.0598	n.s.
		CS:Treatment	F(2,40)=1.439	0.2492	n.s.
	EXT3	CS	F(2,40)=12.22	<0.001	***
		Treatment	F(1,20)=2.908	0.1036	n.s.
		CS:Treatment	F(2,40)=0.5247	0.5958	n.s.
EXT1 EXT2 systemic SHAM	FC	CS	F(1,28)=93.32	<0.001	***
		Treatment	F(1,28)=2.245	0.1452	n.s.
		CS:Treatment	F(1,28)=0.1467	0.7046	n.s.
	EXT1	CS	F(2,28)=40.45	<0.001	***
		Treatment	F(1,14)=8.389e-06	0.9977	n.s.
		CS:Treatment	F(2,28)=0.3898	0.6808	n.s.
	EXT2	CS	F(2,28)=3.663	0.03862	*
		Treatment	F(1,14)=0.1186	0.7357	n.s.
		CS:Treatment	F(2,28)=1.427	0.257	n.s.
	EXT3	CS	F(2,28)=0.3484	0.7088	n.s.
		Treatment	F(1,14)=0.8528	0.3714	n.s.
		CS:Treatment	F(2,28)=0.89	0.4219	n.s.
Local infusion in MD	FC	CS	F(1,28)=180.6	<0.001	***
		Treatment	F(2,28)=3.891	0.03228	*
		CS:Treatment	F(2,28)=0.8224	0.4497	n.s.
	EXT1	CS	F(2,28)=28.78	<0.001	***
		Treatment	F(2,14)=2.142	0.1543	n.s.
		CS:Treatment	F(4,28)=2.719	0.0497	*
	EXT2	CS	F(2,28)=8.082	0.001	**
		Treatment	F(2,14)=0.2967	0.7479	n.s.
		CS:Treatment	F(4,28)=0.7401	0.5726	n.s.
	EXT3	CS	F(2,28)=6.821	0.0038	**
		Treatment	F(2,14)=2.013	0.1705	n.s.
		CS:Treatment	F(4,28)=0.2776	0.89	n.s.
EXT3 systemic	FC	CS	F(1,12)=75.87	<0.001	***
		Treatment	F(1,12)=0.2627	0.6175	n.s.
		CS:Treatment	F(1,12)=3.799	0.07506	n.s.
	EXT1	CS	F(2,24)=38.94	<0.001	***
		Treatment	F(1,12)=0.07624	0.7872	n.s.
		CS:Treatment	F(2,24)=0.3532	0.7061	n.s.
	EXT2	CS	F(2,24)=3.517	0.04576	*
		Treatment	F(1,12)=0.8919	0.3636	n.s.
		CS:Treatment	F(2,24)=2.072	0.1479	n.s.
	EXT3	CS	F(2,24)=0.3027	0.7416	n.s.
		Treatment	F(1,12)=0.007115	0.9342	n.s.
		CS:Treatment	F(2,24)=0.8221	0.4515	n.s.

Supplementary Table 9: Statistics for SupFig 3a-d ANOVAs.

Experiment	Group 1	Group 2	Phase	CS period	Estimate	SE	df	t	p-value	Sig.
EXT1 EXT2 systemic	Gi+CNO	CT+SAL	FC	CS1	5.41	6.52	35.74	0.83	0.41	n.s.
				CS5	-1.18	6.52	35.74	-0.18	0.86	n.s.
			EXT1	Early	0.58	7.44	35.33	0.08	0.94	n.s.
				Middle	-5.50	7.44	35.33	-0.74	0.46	n.s.
				Late	-16.90	7.44	35.33	-2.27	0.03	*
			EXT2	Early	-11.17	10.57	29.90	-1.06	0.30	n.s.
				Middle	-24.00	10.57	29.90	-2.27	0.03	*
				Late	-21.66	10.57	29.90	-2.05	0.05	*
			EXT3	Early	-8.80	7.92	30.63	-1.11	0.28	n.s.
				Middle	-15.16	7.92	30.63	-1.91	0.07	n.s.
				Late	-12.18	7.92	30.63	-1.54	0.13	n.s.
EXT1 EXT2 systemic SHAM	SHAM+CNO	SHAM+SAL	FC	CS1	10.62	7.98	28.00	1.33	0.19	n.s.
				CS5	6.30	7.98	28.00	0.79	0.44	n.s.
			EXT1	Early	4.99	9.62	28.58	0.52	0.61	n.s.
				Middle	-2.14	9.62	28.58	-0.22	0.83	n.s.
				Late	-2.78	9.62	28.58	-0.29	0.77	n.s.
			EXT2	Early	-2.77	10.23	31.52	-0.27	0.79	n.s.
				Middle	-12.12	10.23	31.52	-1.19	0.24	n.s.
				Late	6.68	10.23	31.52	0.65	0.52	n.s.
			EXT3	Early	5.76	8.77	28.79	0.66	0.52	n.s.
				Middle	1.00	8.77	28.79	0.11	0.91	n.s.
				Late	12.88	8.77	28.79	1.47	0.15	n.s.
Local infusion in MD	CT+SAL	Gi+CNO	FC	CS1	13.02	8.02	28.00	1.62	0.25	n.s.
				CS5	18.46	8.02	28.00	2.30	0.07	n.s.
			EXT1	Early	-9.18	8.60	26.25	-1.07	0.54	n.s.
				Middle	-5.64	8.60	26.25	-0.66	0.79	n.s.
				Late	-24.95	8.60	26.25	-2.90	0.02	*
			EXT2	Early	-17.08	12.65	27.10	-1.35	0.38	n.s.
				Middle	-7.57	12.65	27.10	-0.60	0.82	n.s.
				Late	3.72	12.65	27.10	0.29	0.95	n.s.
			EXT3	Early	-10.01	7.76	37.80	-1.29	0.41	n.s.
				Middle	-4.30	7.76	37.80	-0.55	0.84	n.s.
				Late	-10.21	7.76	37.80	-1.32	0.40	n.s.
	CT+SAL	CT+CNO	FC	CS1	1.56	7.64	28.00	0.20	0.98	n.s.
				CS5	15.33	7.64	28.00	2.01	0.13	n.s.
			EXT1	Early	-6.66	8.20	26.25	-0.81	0.70	n.s.
				Middle	9.11	8.20	26.25	1.11	0.52	n.s.
				Late	-3.52	8.20	26.25	-0.43	0.90	n.s.
			EXT2	Early	-11.52	12.06	27.10	-0.96	0.61	n.s.
				Middle	-8.29	12.06	27.10	-0.69	0.77	n.s.
				Late	0.20	12.06	27.10	0.02	1.00	n.s.
			EXT3	Early	-6.96	7.40	37.80	-0.94	0.62	n.s.
				Middle	-10.15	7.40	37.80	-1.37	0.37	n.s.
				Late	-12.18	7.40	37.80	-1.65	0.24	n.s.
	Gi+CNO	CT+CNO	FC	CS1	-11.46	8.02	28.00	-1.43	0.34	n.s.
				CS5	-3.13	8.02	28.00	-0.39	0.92	n.s.
			EXT1	Early	2.51	8.60	26.25	0.29	0.95	n.s.
				Middle	14.74	8.60	26.25	1.71	0.22	n.s.
				Late	21.43	8.60	26.25	2.49	0.05	*
			EXT2	Early	5.57	12.65	27.10	0.44	0.90	n.s.
				Middle	-0.72	12.65	27.10	-0.06	1.00	n.s.
				Late	-3.52	12.65	27.10	-0.28	0.96	n.s.
			EXT3	Early	3.05	7.76	37.80	0.39	0.92	n.s.
				Middle	-5.85	7.76	37.80	-0.75	0.73	n.s.
				Late	-1.98	7.76	37.80	-0.25	0.96	n.s.
EXT3 systemic	CT+SAL	Gi+CNO	FC	CS1	15.19	8.77	23.99	1.73	0.10	n.s.
				CS5	-8.78	8.77	23.99	-1.00	0.33	n.s.
			EXT1	Early	6.54	9.70	26.38	0.67	0.51	n.s.
				Middle	1.95	9.70	26.38	0.20	0.84	n.s.
				Late	-2.18	9.70	26.38	-0.22	0.82	n.s.
			EXT2	Early	5.69	11.22	26.84	0.51	0.62	n.s.
				Middle	-12.62	11.22	26.84	-1.12	0.27	n.s.
				Late	-17.87	11.22	26.84	-1.59	0.12	n.s.
			EXT3	Early	6.78	10.24	23.75	0.66	0.51	n.s.
				Middle	-3.79	10.24	23.75	-0.37	0.71	n.s.
				Late	-5.11	10.24	23.75	-0.50	0.62	n.s.

Supplementary Table 10: Statistics for SupFig 3a-d contrasts.

Experiment	Effect	ANOVA	p-value	Sig.
EXT1 EXT2 systemic	Group	F(1,20)=1.315,	0.27	n.s.
	Context	F(3,54)=62.02,	0.00	***
	Group:Context	F(3,54)=0.3499,	0.79	n.s.
EXT1 EXT2 systemic SHAM	Group	F(2,14)=0.5874,	0.57	n.s.
	Context	F(3,42)=25.29,	0.00	***
	Group:Context	F(6,42)=0.7821,	0.59	n.s.
Local infusion in MD	Group	F(1,14)=0.147,	0.71	n.s.
	Context	F(3,42)=22.59,	0.00	***
	Group:Context	F(3,42)=0.6034,	0.62	n.s.
EXT3 systemic	Group	F(1,10)=1.364,	0.27	n.s.
	Context	F(3,30)=64.24,	0.00	***
	Group:Context	F(3,30)=3.188,	0.04	*

Supplementary Table 11: Statistics for SupFig 3e-h ANOVAs.

group	context	Group 1	Group 2	Estimate	SE	df	t.ratio	p.value	Sig.
EXT1 EXT2 systemic	CONTEXT A	CT+SAL	Gi+CNO	3.81	4.01	20.00	0.95	0.35	n.s.
	CONTEXT B preEXT1	CT+SAL	Gi+CNO	1.76	4.01	20.00	0.44	0.67	n.s.
	CONTEXT B preEXT2	CT+SAL	Gi+CNO	4.15	4.19	20.00	0.99	0.33	n.s.
	CONTEXT B preEXT3	CT+SAL	Gi+CNO	7.38	4.19	20.00	1.76	0.09	n.s.
EXT1 EXT2 systemic SHAM	CONTEXT A	SHAM+SAL	SHAM+CNO	0.71	5.90	14.00	0.12	0.91	n.s.
	CONTEXT B preEXT1	SHAM+SAL	SHAM+CNO	-2.78	5.90	14.00	-0.47	0.64	n.s.
	CONTEXT B preEXT2	SHAM+SAL	SHAM+CNO	5.54	5.90	14.00	0.94	0.36	n.s.
	CONTEXT B preEXT3	SHAM+SAL	SHAM+CNO	3.21	5.90	14.00	0.54	0.59	n.s.
Local infusion in MD	CONTEXT A	Gi+CNO	Gi+SAL	-8.00	8.62	14.00	-0.93	0.63	n.s.
	CONTEXT A	Gi+CNO	SHAM+CNO	-4.41	8.62	14.00	-0.51	0.87	n.s.
	CONTEXT A	Gi+SAL	SHAM+CNO	3.59	8.22	14.00	0.44	0.90	n.s.
	CONTEXT B preEXT1	Gi+CNO	Gi+SAL	0.05	8.62	14.00	0.01	1.00	n.s.
	CONTEXT B preEXT1	Gi+CNO	SHAM+CNO	2.29	8.62	14.00	0.27	0.96	n.s.
	CONTEXT B preEXT1	Gi+SAL	SHAM+CNO	2.24	8.22	14.00	0.27	0.96	n.s.
	CONTEXT B preEXT2	Gi+CNO	Gi+SAL	-12.61	8.62	14.00	-1.46	0.34	n.s.
	CONTEXT B preEXT2	Gi+CNO	SHAM+CNO	-8.00	8.62	14.00	-0.93	0.63	n.s.
	CONTEXT B preEXT2	Gi+SAL	SHAM+CNO	4.62	8.22	14.00	0.56	0.84	n.s.
	CONTEXT B preEXT3	Gi+CNO	Gi+SAL	-7.09	8.62	14.00	-0.82	0.70	n.s.
	CONTEXT B preEXT3	Gi+CNO	SHAM+CNO	-12.72	8.62	14.00	-1.48	0.33	n.s.
	CONTEXT B preEXT3	Gi+SAL	SHAM+CNO	-5.63	8.22	14.00	-0.68	0.78	n.s.
EXT3 systemic	CONTEXT A	CT+SAL	Gi+CNO	1.23	4.46	10.00	0.28	0.79	n.s.
	CONTEXT B preEXT1	CT+SAL	Gi+CNO	10.81	4.46	10.00	2.42	0.04	*
	CONTEXT B preEXT2	CT+SAL	Gi+CNO	6.62	4.46	10.00	1.48	0.17	n.s.
	CONTEXT B preEXT3	CT+SAL	Gi+CNO	-2.90	4.46	10.00	-0.65	0.53	n.s.

Supplementary Table 12: Statistics for SupFig3e-h Contrasts.

Parameter	Kruskal	p-value	Sig.	Group1	Group2	p-value	Sig.
Total Distance	1.04	0.59	n.s.	CT+SAL	Gi+CNO	0.94	n.s.
Entries Central Area	3.37	0.18	n.s.	CT+SAL	Gi+CNO	0.21	n.s.
Percentage time Central Area	4.61	0.10	n.s.	CT+SAL	Gi+CNO	0.10	n.s.
Distance moved Central Area	5.30	0.07	n.s.	CT+SAL	Gi+CNO	0.06	n.s.

Supplementary Table 13: Statistics for SupFig 4a.

Parameter	Kruskal	p-value	Sig.	Group1	Group2	p-value	Sig.
Distance moved	0.71	0.70	n.s.	CT+SAL	Gi+CNO	0.83	n.s.
Frequency open arms	0.79	0.67	n.s.	CT+SAL	Gi+CNO	0.82	n.s.
Percentage time opened arms	1.16	0.56	n.s.	CT+SAL	Gi+CNO	0.70	n.s.
Frequency closed arms	2.51	0.28	n.s.	CT+SAL	Gi+CNO	0.64	n.s.

Supplementary Table 14: Statistics for SupFig 4b.

Parameter	Kruskal	p-value	Sig.	Group1	Group2	p-value	Sig.
Frequency Light zone	1.71	0.43	n.s.	CT+SAL	Gi+CNO	0.96	n.s.
Percentage time Light zone	1.67	0.43	n.s.	CT+SAL	Gi+CNO	0.48	n.s.
Latency to enter Dark Zone	0.28	0.87	n.s.	CT+SAL	Gi+CNO	0.95	n.s.

Supplementary Table 15: Statistics for SupFig 4c.

Parameter	Kruskal	p-value	Sig.	Group1	Group2	p-value	Sig.
Hot plate Latency	1.37	0.50	n.s.	CT+SAL	Gi+CNO	0.64	n.s.
Tail immersion Latency	0.03	0.98	n.s.	CT+SAL	Gi+CNO	1.00	n.s.

Supplementary Table 16: Statistics for SupFig 4d.

Parameter	Phase	Group 1	Group 2	Test	Stat	p-value	Sig.
Bursts per second baseline	FC	CT+SAL	Gi+CNO	Mann Whitney U	698.00	0.10	n.s.
	EXT1	CT+SAL	Gi+CNO	Mann Whitney U	819.00	0.00	**
	EXT3	CT+SAL	Gi+CNO	Mann Whitney U	440.00	0.10	n.s.
Bursts per second CS	FC	CT+SAL	Gi+CNO	Mann Whitney U	771.00	0.16	n.s.
	EXT1	CT+SAL	Gi+CNO	Mann Whitney U	899.50	0.01	*
	EXT3	CT+SAL	Gi+CNO	Mann Whitney U	439.00	0.14	n.s.
Average burst firing rate baseline	FC	CT+SAL	Gi+CNO	Mann Whitney U	697.00	0.14	n.s.
	EXT1	CT+SAL	Gi+CNO	Mann Whitney U	1154.00	0.29	n.s.
	EXT3	CT+SAL	Gi+CNO	Mann Whitney U	504.00	0.29	n.s.
Average burst firing rate CS	FC	CT+SAL	Gi+CNO	Mann Whitney U	733.00	0.25	n.s.
	EXT1	CT+SAL	Gi+CNO	Mann Whitney U	1145.00	0.27	n.s.
	EXT3	CT+SAL	Gi+CNO	Mann Whitney U	547.00	0.43	n.s.

Supplementary Table 17: Statistics for Fig 5.

Parameter	Group	Phase 1	Phase 2	Test	Stat	p-value	Sig.
Bursts per second baseline	CT+SAL	FC	EXT1	Mann Whitney U	625.00	0.208	n.s.
		EXT1	EXT3	Mann Whitney U	128.00	<0.001	###
	Gi+CNO	FC	EXT3	Mann Whitney U	904.00	<0.001	###
		EXT1	EXT3	Mann Whitney U	613.00	<0.001	###
Bursts per second CS	CT+SAL	FC	EXT1	Mann Whitney U	539.00	0.803	n.s.
		EXT1	EXT3	Mann Whitney U	197.00	<0.001	###
	Gi+CNO	FC	EXT3	Mann Whitney U	1042.50	<0.001	###
		EXT1	EXT3	Mann Whitney U	1121.00	<0.001	###
Average burst firing rate baseline	CT+SAL	FC	EXT1	Mann Whitney U	501.00	0.018	#
		EXT1	EXT3	Mann Whitney U	162.00	<0.001	###
	Gi+CNO	FC	EXT3	Mann Whitney U	712.00	<0.001	###
		EXT1	EXT3	Mann Whitney U	586.00	<0.001	###
Average burst firing rate CS	CT+SAL	FC	EXT1	Mann Whitney U	478.00	0.018	#
		EXT1	EXT3	Mann Whitney U	163.00	<0.001	###
	Gi+CNO	FC	EXT3	Mann Whitney U	779.00	<0.001	###
		EXT1	EXT3	Mann Whitney U	650.00	<0.001	###

Supplementary Table 18: Statistics for Fig 5 bis.

Variable	Group	Test	Condition 1	Condition 2	Statistic	p-value	Sig.
Fraction of PSD	CT+SAL	Wilcoxon	Baseline	CS	0.00	<0.001	***
	Gi+CNO	Wilcoxon	Baseline	CS	1.00	<0.001	***

Supplementary Table 19: Statistics for Fig 6c.

Variable	Condition	Test	Condition 1	Condition 2	Statistic	p-value	Sig.
Fraction of PSD	CS	Mann Whitney U	CT+SAL	Gi+CNO	22.00	0.00	##

Supplementary Table 20: Statistics for Fig 6c bis.

Group	Test	Statistic	p-value	Sig.
CT+SAL during CS	One sample Wilcoxon test	100.00	<0.001	***
Gi+CNO during CS	One sample Wilcoxon test	460.00	<0.001	***
CT+SAL between CSs	One sample Wilcoxon test	212.00	0.0129	*
Gi+CNO between CSs	One sample Wilcoxon test	467.00	<0.001	***

Supplementary Table 21: Statistics for Fig 6f.

Condition 1	Condition 2	Test	Statistic	p-value	Sig.
CT+SAL during CS	Gi+CNO during CS	Mann Whitney U	1013.00	0.0274	#
CT+SAL between CSs	Gi+CNO between CSs	Mann Whitney U	1295.00	0.4712	n.s.
CT+SAL during CS	CT+SAL between CSs	Wilcoxon	136.00	<0.001	***
Gi+CNO during CS	Gi+CNO between CSs	Wilcoxon	990.00	0.3519	n.s.

Supplementary Table 22: Statistics for Fig 6f bis.

Variable	Test	Condition 1	Condition 2	Statistic	p-value	Sig.
Burst occurence	Mann Whitney U	CT+SAL	Gi+CNO	890.00	0.00	##

Supplementary Table 23: Statistics for Fig 6g.

Value	Test	Group	Z	p-value	Sig.
MD bursting phase	Rayleigh test	CT+SAL	5.67	0.0034	**
	Rayleigh test	Gi+CNO	90.56	<0.001	***

Supplementary Table 24: Statistics for Fig 7b.

Value	Test	Group	Z	p-value	Sig.
MD neurons preferential bursting phase	Rayleigh test	CT+SAL	4.67	0.0085	**
	Circular V-test (dir=pi)	CT+SAL	12.50	0.0023	##
	Rayleigh test	Gi+CNO	17.98	<0.001	***
	Circular V-test (dir=pi)	Gi+CNO	33.94	<0.001	###

Supplementary Table 25: Statistics for Fig 7c.

Value	Category	Test	Group	Statistic	p-value	Sig.
Average dmPFC 4Hz relative to MD bursting (prior vs post bursting)	High 4Hz episode	Wilcoxon	CT+SAL	35.00	<0.001	***
		Wilcoxon	Gi+CNO	143.00	<0.001	***

Supplementary Table 26: Statistics for Fig 7d.

Value	Test	Group	Statistic	p-value	Sig.
Ratio Post/Pre-burst 4Hz amplitude (low vs high 4Hz episode)	Wilcoxon	CT+SAL	31.00	<0.001	***
	Wilcoxon	Gi+CNO	215.00	<0.001	***

Supplementary Table 27: Statistics for Fig 7e.

Variable	Test	Condition 1	Condition 2	Statistic	p-value	Sig.
Angular distance	Mann Whitney U	CT+SAL	Gi+CNO	973.00	0.01	#

Supplementary Table 28: Statistics for SupFig 5a.

Value	Category	Test	Group	Statistic	p-value	Sig.
Average dmPFC 4Hz relative to MD bursting (prior vs post bursting)	Low 4Hz episode	Wilcoxon	CT+SAL	338.00	0.47	n.s.
		Wilcoxon	Gi+CNO	953.00	0.25	n.s.

Supplementary Table 29: Statistics for SupFig 5b.

Variable	Group	Test	Condition 1	Condition 2	Statistic	p-value	Sig.
Fraction of PSD	CT+SAL	Wilcoxon	Baseline	CS	0.00	<0.001	***
	Gi+CNO	Wilcoxon	Baseline	CS	68.00	0.0326	*

Supplementary Table 30: Statistics for Fig 8c.

Variable	Group	Test	Statistic	p-value	Sig.
Lag of peak cross-correlation	CT+SAL	Wilcoxon	456.00	<0.001	***
dmPFC - MD 4Hz LFP	Gi+CNO	Wilcoxon	351.00	<0.001	***

Supplementary Table 31: Statistics for Fig 8e.

Variable	Group	Test	Direction 1	Direction 2	Statistic	p-value	Sig.
4Hz range GPDC EXT1	CT+SAL	Wilcoxon	dmPFC->MD	MD->dmPFC	87.00	<0.001	***
	Gi+CNO	Wilcoxon	dmPFC->MD	MD->dmPFC	925.00	<0.001	***

Supplementary Table 32: Statistics for Fig 8f.

Value	Test	Group 1	Group 2	Statistic	p-value	Sig.
4Hz GPDC dmPFC->MD	Mann Whitney U	CT+SAL	Gi+CNO	8045.00	<0.001	***
4Hz GPDC MD->dmPFC	Mann Whitney U	CT+SAL	Gi+CNO	5894.00	<0.001	***

Supplementary Table 33: Statistics for Fig 8g.

Value	Test	Group 1	Group 2	Statistic	p-value	Sig.
Basal dmPFC-MD 4Hz coherence before MD bursting	Mann Whitney U	CT+SAL	Gi+CNO	809.00	<0.001	***

Supplementary Table 34: Statistics for Fig 9c.

Test	Group	Value 1	Value 2	Statistic	p-value	Sig.
Wilcoxon test	CT+SAL	4HzCohbasal	4HzCoh 1st cycle before burst	98.00	<0.001	***
		4HzCohbasal	4HzCoh 1st cycle after burst	95.00	<0.001	***
		4HzCohbasal	4HzCoh 2nd cycle after burst	223.00	0.0197	*
	Gi+SAL	4HzCohbasal	4HzCoh 1st cycle before burst	366.00	<0.001	***
		4HzCohbasal	4HzCoh 1st cycle after burst	247.00	<0.001	***
		4HzCohbasal	4HzCoh 2nd cycle after burst	842.00	0.0635	n.s.

Supplementary Table 35: Statistics for Fig 9d.

Test	Group	Value 1	Value 2	Statistic	p-value	Sig.
Wilcoxon test	CT+SAL	4HzCoh 1st cycle before burst	4HzCoh 1st cycle after burst	202.00	0.01	##
	Gi+SAL	4HzCoh 1st cycle before burst	4HzCoh 1st cycle after burst	761.00	0.02	#

Supplementary Table 36: Statistics for Fig 9d bis.
