# Supplementary material for: The cerebellum regulates fear extinction through thalamo-prefrontal cortex interactions in male mice
Source: Nat Commun. 2023 Mar 17;14:1508. doi: 10.1038/s41467-023-36943-w (PMC10023697; doi:10.1038/s41467-023-36943-w)
Supplement: Supplementary file 3 — Reporting Summary [file 41467_2023_36943_MOESM3_ESM.pdf]

nature portfolio

Corresponding author(s): Clément Léna

Last updated by author(s): 2022/12/12

Reporting Summary

Nature Portfolio wishes to improve the reproducibility of the work that we publish. This form provides structure for consistency and transparency in reporting. For further information on Nature Portfolio policies, see our Editorial Policies and the Editorial Policy Checklist.

Statistics

For all statistical analyses, confirm that the following items are present in the figure legend, table legend, main text, or Methods section.

n/a	Confirmed	
☐	☒	The exact sample size (*n*) for each experimental group/condition, given as a discrete number and unit of measurement
☐	☒	A statement on whether measurements were taken from distinct samples or whether the same sample was measured repeatedly
☐	☒	The statistical test(s) used AND whether they are one- or two-sided<br>*Only common tests should be described solely by name; describe more complex techniques in the Methods section.*
☐	☒	A description of all covariates tested
☐	☒	A description of any assumptions or corrections, such as tests of normality and adjustment for multiple comparisons
☐	☒	A full description of the statistical parameters including central tendency (e.g. means) or other basic estimates (e.g. regression coefficient) AND variation (e.g. standard deviation) or associated estimates of uncertainty (e.g. confidence intervals)
☐	☒	For null hypothesis testing, the test statistic (e.g. *F*, *t*, *r*) with confidence intervals, effect sizes, degrees of freedom and *P* value noted<br>*Give P values as exact values whenever suitable.*
☒	☐	For Bayesian analysis, information on the choice of priors and Markov chain Monte Carlo settings
☐	☒	For hierarchical and complex designs, identification of the appropriate level for tests and full reporting of outcomes
☐	☒	Estimates of effect sizes (e.g. Cohen's *d*, Pearson's *r*), indicating how they were calculated

Our web collection on statistics for biologists contains articles on many of the points above.

Software and code

Policy information about availability of computer code

Data collection	The electrophysiological recordings were performed with Multi-Channel System W2100 system and their proprietary software multichannel (v1.5.6) Tucker-Davis-Technologies System using their proprietary Synapse software version 95.<br>The pictures were acquired on Leica TCS Sp8<br>The behavioral experiments were controlled by Ethovision X14.
Data analysis	Prism 7, R 3.6.3 with packages lme 3.1-3 and emmeans 1.7.0 , Rstudio 4.1.2, Ethovision XT14

For manuscripts utilizing custom algorithms or software that are central to the research but not yet described in published literature, software must be made available to editors and reviewers. We strongly encourage code deposition in a community repository (e.g. GitHub). See the Nature Portfolio guidelines for submitting code & software for further information.

Data

Policy information about availability of data

All manuscripts must include a data availability statement. This statement should provide the following information, where applicable:

- Accession codes, unique identifiers, or web links for publicly available datasets
- A description of any restrictions on data availability
- For clinical datasets or third party data, please ensure that the statement adheres to our policy

The data generated during this study have been deposited on the Dryad database with the reference doi:10.5061/dryad.9kd51c5ng

The source code generated during this study have been deposited on the Dryad database with the reference https://doi.org/10.5281/zenodo.7603770

Human research participants

Policy information about studies involving human research participants and Sex and Gender in Research.

Reporting on sex and gender	NA
Population characteristics	NA
Recruitment	NA
Ethics oversight	NA

Note that full information on the approval of the study protocol must also be provided in the manuscript.

Field-specific reporting

Please select the one below that is the best fit for your research. If you are not sure, read the appropriate sections before making your selection.

☒ Life sciences ☐ Behavioural & social sciences ☐ Ecological, evolutionary & environmental sciences

For a reference copy of the document with all sections, see nature.com/documents/nr-reporting-summary-flat.pdf

Life sciences study design

All studies must disclose on these points even when the disclosure is negative.

Sample size	Our sample-size are consistent with usual sample size's in the literature (Wehner JM, Radcliffe RA. Curr Protoc Neurosci. 2004 Chapter 8). Sample size was confirmed to be adequate based on the magnitude and consistency of measurable differences between groups. The sample size (n) of each experiment is provided in the corresponding figure captions in the main manuscript and in materials and methods section. Sample sizes were chosen to support meaningful conclusions in accordance with ethical committee requirements to limit as much as possible the use of animals.
Data exclusions	If postmortem analysis indicated misplacement of electrodes, the data from the corresonding region were removed from the analysis.
Replication	Each experiment presented in this paper was repeated in multiple series of animals (3-5 times). Each series contained animals assigned to different groups. The observations from each group thus derive from multiple independent successful experiments.
Randomization	Animals were assigned randomly to experimental and control groups.
Blinding	The behavioral experiments were automatized; the experimenters were not blinded to the drugs injected for practical reasons (experimenters worked alone). Group allocation was otherwise blind to the experimenters and during data curation (spike sorting).

Reporting for specific materials, systems and methods

We require information from authors about some types of materials, experimental systems and methods used in many studies. Here, indicate whether each material, system or method listed is relevant to your study. If you are not sure if a list item applies to your research, read the appropriate section before selecting a response.

Materials & experimental systems

n/a	Involved in the study	
☐	☒	Antibodies
☒	☐	Eukaryotic cell lines
☒	☐	Palaeontology and archaeology
☐	☒	Animals and other organisms
☒	☐	Clinical data
☒	☐	Dual use research of concern

Methods

n/a	Involved in the study	
☒	☐	ChIP-seq
☒	☐	Flow cytometry
☒	☐	MRI-based neuroimaging

Antibodies

Antibodies used	anti-Calbindin-D-28K (1:300, Sigma, C9848)<br>donkey anti-mouse IgG conjugated to Alexa Fluor 488 (1:300, Invitrogen, R37114)
Validation	Ohm T G, et al. Neuroscience, 42, 823 -823 (1991)<br>Katsetos C D, et al. Archives of Pathology & Laboratory Medicine, 118, 633 -633 (1994)<br>L M Garcia-Segura et al. Brain research, 296(1), 75-86 (1984-03-26)

Animals and other research organisms

Policy information about studies involving animals; ARRIVE guidelines recommended for reporting animal research, and Sex and Gender in Research

Laboratory animals	Adult male C57BL/6N mice 8-12 weeks old (Charles River, France,MSR Cat# CRL_27,RRID:IMSR_CRL:27)
Wild animals	NA
Reporting on sex	Adult male wild-type mice 8 - 12 weeks of age were used for all the experiments. Males and females mice are equally suitable for fear conditioning analysis 71, but male mice are bigger and have therefore more strength to carry the implant and preamplifier in electrophysiological experiments.
Field-collected samples	NA
Ethics oversight	Animal care and experimental procedures followed the European Community Council Directives (authorization number APAFIS #29793-202102121752192 v3 & APAFIS #1334-2015070818367911)

Note that full information on the approval of the study protocol must also be provided in the manuscript.
